# Human Papillomavirus Is Rare and Does Not Correlate with p16^INK4A^ Expression in Non-Small-Cell Lung Cancer in a Jordanian Subpopulation

**DOI:** 10.3390/medicina60040660

**Published:** 2024-04-19

**Authors:** Ola Abu Al Karsaneh, Arwa Al Anber, Sahar AlMustafa, Hussien AlMa’aitah, Batool AlQadri, Abir Igbaria, Rama Tayem, Mustafa Khasawneh, Shaima Batayha, Tareq Saleh, Mohammad ALQudah, Maher Sughayer

**Affiliations:** 1 Department of Microbiology, Pathology and Forensic Medicine, Faculty of Medicine, The Hashemite University, Zarqa 13133, Jordan; m.alqudah12@hu.edu.jo; 2 Department of Pharmacology and Public Health, Faculty of Medicine, The Hashemite University, Zarqa 13133, Jordan; arwaa@hu.edu.jo (A.A.A.); tareq@hu.edu.jo (T.S.); 3 Department of Pathology and Laboratory Medicine, King Hussein Cancer Center, Amman 11941, Jordan; sa.15353@khcc.jo (S.A.); ha.11789@khcc.jo (H.A.); 4 Faculty of Medicine, Jordan University of Science and Technology, Irbid 22110, Jordan; bnalqadri17@med.just.edu.jo (B.A.); aaigbaria17@med.just.edu.jo (A.I.); rmtayem17@med.just.edu.jo (R.T.); mqkhasawneh171@med.just.edu.jo (M.K.); 5 Department of Pathology and Microbiology, Faculty of Medicine, Jordan University of Science and Technology, Irbid 22110, Jordan; sabatayha20@med.just.edu.jo

**Keywords:** HPV, PCR, p16^INK4a^, immunohistochemistry, NSCLC, Jordan

## Abstract

*Background and Objectives*: Human papillomavirus (HPV) was previously investigated in lung cancer with wide inter-geographic discrepancies. p16^INK4a^ has been used as a surrogate for detecting high-risk HPV (HR-HPV) in some cancer types. This study assessed the evidence of HPV in non-small-cell lung cancer (NSCLC) among Jordanian patients, investigated the expression of p16^INK4a^, and evaluated its prognostic value and association with HPV status. *Materials and Methods*: The archived samples of 100 patients were used. HPV DNA detection was performed by real-time polymerase chain reaction (RT-PCR). p16^INK4a^ expression was assessed by immunohistochemistry (IHC). The Eighth American Joint Committee on Cancer protocol (AJCC) of head and neck cancer criteria were applied to evaluate p16^INK4a^ positivity considering a moderate/strong nuclear/cytoplasmic expression intensity with a distribution in ≥75% of cells as positive. *Results*: HPV DNA was detected in 5% of NSCLC cases. Three positive cases showed HR-HPV subtypes (16, 18, 52), and two cases showed the probable HR-HPV 26 subtype. p16^INK4a^ expression was positive in 20 (20%) NSCLC cases. None of the HPV-positive tumors were positive for p16^INK4a^ expression. A statistically significant association was identified between p16^INK4a^ expression and the pathological stage (*p =* 0.029) but not with other variables. No survival impact of p16^INK4a^ expression was detected in NSCLC cases as a group; however, it showed a statistically significant association with overall survival (OS) in squamous cell carcinoma (SqCC) cases (*p* = 0.033). *Conclusions*: This is the first study to assess HPV and p16^INK4a^ expression in a Jordanian population. HPV positivity is rare in NSCLC among a Jordanian subpopulation. P16 ^INK4a^ reliability as a surrogate marker for HPV infection in lung cancer must be revisited.

## 1. Introduction

Lung cancer is the leading cause of cancer-related morbidity and mortality in males and females worldwide [1]. Lung cancer pathogenesis is a complex process involving genetic and environmental factors [2]. Although smoking is the most significant causative agent for lung cancer, fewer than 20% of smokers develop lung cancer, and lung cancer remains the primary cause of death among never-smokers [3,4]. Other factors, such as genetic susceptibility, unfavorable occupational exposure, air pollution, and viral infections, are implicated in the development of lung cancer [5,6]. Human papillomavirus (HPV) is one of the viruses associated with lung cancer [7].

HPV is a DNA virus that belongs to the Papovaviridae family and is genotyped into three groups based on its oncogenic risk: high-risk group (HR-HPV) such as HPVs 16, 18, 31, 33, 35, 39, 45, 51, and 52, which can lead to cancerous transformation, probable/possible carcinogen such as HPVs 26, 53, 66, 68, 73, and 82, and low-risk- group (LR-HPV) such as HPVs 6, 11, 40, 42, 43, 44, 54, 61, and 72, which cause benign lesions [8]. The HPV DNA encodes for several proteins, including the early (E) E1-E2, E4-E7, and late (L), L1, and L2 proteins, that carry oncogenic potential [9].

The role of HPV in causing cervical cancer and a subset of anogenital and oropharyngeal cancers is established [10,11,12]. In this regard, a recent study detected a high frequency of HPV DNA (94.4%) in high-grade vaginal intraepithelial neoplasia, with 26.8% showing multiple infections [13]. Furthermore, these HPV-induced cancers show overexpression of the p16^INK4a^ protein, which is used as a surrogate for HPV status in these cancers [14,15]. The molecular pathways of HPV-induced cervical and head and neck carcinomas are linked to its two main oncogenes, E6 and E7, which deactivate the p53 and Rb tumor suppressor genes, respectively. After infection, the HPV-E7 protein binds to the Rb protein, causing its inactivation and leading to the release of E2F, which drives the expression of several pro-proliferative proteins. Several studies showed that Rb inactivation and the consequent E2F activation stimulate the expression of the tumor suppressor gene p16^INK4a^, a cyclin-dependent kinase inhibitor (CDKI), through negative feedback [16,17,18]. Subsequently, p16^INK4a^ expression has been highly correlated with HR-HPV.

On the other hand, although several studies have also indicated that HR-HPV may have a role in the pathogenesis of lung cancer [2,7,19], this role has not been well established. The first report to suggest a connection between HPV and lung cancer was in 1979 by Syrjaanen et al., indicating the presence of histological changes similar to the HPV-linked condylomatous changes in the bronchial epithelium adjacent to squamous cell carcinoma [20]. Since then, several studies have been conducted and showed conflicting results regarding the relationship between HPV infection and lung cancer development, with inconsistency in the reported prevalence, possibly due to geographic differences and methodological variability. For example, in one meta-analysis, Srinivasan et al. found that the prevalence of HPV varied greatly between geographic areas and histological subtypes, with a global range of 0.0 to 78.3%. This meta-analysis, in addition, showed that Asian studies reported a higher HPV prevalence compared to European studies [4]. Unlike cervical and head and neck cancers, the relationship between HPV and p16^INK4a^ expression in NSCLC is not well established. Some studies failed to reveal a significant correlation between HPV infection and p16^INK4a^ expression in lung cancer [15,21]. In any case, p16^INK4a^ expression has been evaluated for its prognostic role in NSCLC regardless of the HPV status, and several studies showed that it had a significant prognostic value [22,23,24,25].

In Jordan, lung cancer is the leading cause of cancer-related deaths, and recent estimates expect an increasing burden of lung cancer on public health, particularly with increasing smoking habits. Further, the prevalence of HPV, despite being relatively low, seems to be increasing in Jordan [26]. However, to the best of our knowledge, there is no data that link lung cancer and HPV among Jordanian patients. This study aimed to evaluate the prevalence of HPV and p16^INK4a^ expression in NSCLC among a Jordanian subpopulation.

## 2. Materials and Methods

### 2.1. Patients and Tissue Samples

A total of 100 tumor tissue samples of patients who were surgically treated for their NSCLC between 2009 and 2022 at King Hussein Cancer Center (KHCC), Amman, Jordan, were obtained for this work. The cases were selected after a retrospective search of the archived cases at the Department of Pathology and Laboratory Medicine, KHCC. The patients’ inclusion criteria included the following: patients with surgical resection of primary lung cancer, lack of preoperative chemotherapy or radiotherapy, negative history of other tumors at the time of diagnosis, particularly those derived from an HPV-driven anatomical area, and the presence of adequate tissue samples and clinical follow-up data. The patients’ clinicopathological characteristics, including age, gender, smoking status, postoperative treatment history, histologic subtypes, pathologic stage, and survival data, were collected from the patients’ medical records and pathological reports. The respective H&E-stained slides of the cases were evaluated by two pathologists (O.A.A.K. and S.A.) to confirm the pathological features. Tumor subtypes and grades were determined according to the World Health Organization (WHO) guidelines [27,28]. Grading of the adenocarcinoma (ADC) cases was determined based on the combination of the predominant and the worst architectural patterns. Squamous cell carcinoma (SqCC) grades were specified based on the degree of tumor keratinization. Tumor grades were divided into two categories: low-grade, which included well to moderately differentiated cancers, and high-grade, which included cancers with poor differentiation. The stages were determined according to the seventh and eighth editions of the American Joint Committee (AJC) on the Cancer TNM classification system, depending on the time of diagnosis [29,30]. Overall survival (OS) was measured from the time of surgery to the time of death or the last follow-up visit. Disease-free survival (DFS) was calculated from the time of surgery to the time of the first of two events: recurrence or death from any cause.

### 2.2. Extraction of DNA

FFPE tissue blocks were retrieved from the pathology department in KHCC. For each patient, 10 sections of 10 μm thickness were cut using the HistoCore BIOCUT manual rotary microtome, where the blade gently sweeps the sections after cutting to be stored in 2 mL microcentrifuge tubes positioned underneath. Once all sections had been collected, the tube was securely capped to prevent sample loss or contamination. DNA extraction was carried out using the ReliaPrep™ FFPE gDNA Miniprep System^®^ Genomic DNA Kits (Promega, Madison, WI, USA) according to the manufacturer’s instructions. Briefly, the FFPE sections were deparaffinized by adding 1 mL of 100% xylene to the samples with centrifuging for 2 min at maximum speed at room temperature to remove residual xylene, then adding 1 mL of 95–100% ethanol (100%) with centrifuging for 2 min at maximum speed at room temperature. After that, centrifuging for 30 s at maximum speed and drying the pellet for 5–15 min at 37 °C was performed to evaporate residual ethanol. Then, 200 µL of Lysis Buffer and 20 µL of Proteinase K were directly added to the samples and incubated at 56 °C for 1 h and at 80 °C for 4 h. Subsequently, 10 μL of RNAse A was added to the lysed sample and incubated at room temperature for 5 min. Then, 220 µL of BL Buffer and 240 µL of ethanol (95–100%) were added and mixed thoroughly, followed by the binding and column washing steps. Then, DNA was eluted by adding 50 µL of Elution Buffer, centrifuging at 16,000× *g* for 1 min, and storing at −30 to −10 °C for subsequent processing.

### 2.3. HPV Detection and Genotyping

HPV detection and genotyping were performed by real-time PCR (RT-PCR) using the HPV Genotypes 21 Real-TM Quant Kit (Sacace Biotechnologies, Como, Italy). The HPV Genotypes 21 Real-TM Quant Kit is capable of identifying 21 HPV subtypes, including three low-risk (HPV 6, 11, 44), six probable high-risk (HPV 26, 53, 66, 68, 73, 82), and 12 high-risk (HPV 16, 18, 31, 33, 35, 39, 45, 51, 52, 56, 58, 59). The kit amplifies a target of DNA sequence. The kit contains an artificial sequence of the HPV genome as a positive internal control (IC) to monitor the quality and efficiency of the PCR and the DNA amplification process and a PCR mix for the amplification of human genomic DNA (sample intake control (SIC)) that allows for the exclusion of preanalytical error. The kit also provides positive and negative controls. Briefly, 10.0 μL of Taq DNA Polymerase was added to each strip of PCR-mix 21 along with 5.0 μL of extracted DNA from the clinical samples. The negative and positive control strips received the same mix. After that, all strips were capped and spun for 2–3 s and transferred into the thermal cycler (Anatolia geneworks RT-PCR) and went through the following program: 1 cycle of 80 °C for 30 s followed by 94 °C for 90 s; 5 cycles of 94 °C for 30 s followed by 64 °C for 15 s; 45 cycles of 94 °C for 10 s followed by 64 °C for 25 s; and finally 1 cycle of 94 °C for 5 s. The PCR was considered valid if the positive controls showed a signal and the negative controls did not. Results interpretation was performed using the software of SLAN 8.3 RT-PCR system.

### 2.4. Immunohistochemistry (IHC)

The specimens were fixed in 10% neutral-buffered formalin (NBF) immediately or within 5 min of the resection, with a fixation duration between 12 and 28 h. Immunohistochemistry was performed using a BOND III autostainer and Detection Kit (DS9800; Leica Biosystems, Deer Park, TX, USA) according to the manufacturer’s instructions. Briefly, tissue sections of 3 μm thickness were cut from the FFPE specimens using the HistoCore BIOCUT manual rotary microtome, prepared on IHC adhesive slides (TOMO^®^), and put in an oven for about 15 min at 65–70 °C to assist the sections adhering to the slides. Then, the sections were deparaffinized and rehydrated. Antigen retrieval was performed using Bond Epitope Retrieval Solution 1 (Citrate; pH 6.0) (AR9961; Leica Biosystems) for 20 min. Blocking was achieved with Peroxide 3–4% (vol/vol) hydrogen peroxide for 5 min at 100 °C. Slides were incubated at room temperature with the ready-to-use anti-p16 mouse monoclonal primary antibody for 20 min (clone INK4A/JC2, Cell Marque, Rocklin, CA, USA). Visualization was achieved using 3,3′-diaminobenzidine (DAB) staining and hematoxylin counterstaining (Leica Biosystems). Finally, the slides were dehydrated and cleared with xylene, then cover-slipped with dibutyl phthalate in xylene (D.P.X.) mounting media. Cervical squamous severe dysplasia known to be p16-positive was used as a positive control, and negative control was obtained by removing the primary antibody.

### 2.5. p16^INK4a^ Protein Expression Scoring

The samples were assessed and scored independently by two pathologists (O.A.A.K. and S.A.) using a light microscope (Olympus BX53, Tokyo, Japan), and a consensus was obtained on all cases. All cases were scored for staining intensity in tumor cells as follows: 0: unstained, 1+: weak, 2+: moderate, 3+: strong. The distribution of staining in tumor cells was scored as follows: 0: no staining, 1+: 1–<25%, 2+: 25–<50%, 3+: 50–<75%, 4+: ≥75%. Then, following the 8th edition of the AJCC staging system for head and neck cancer, a positive p16^INK4a^ expression was defined as cases with nuclear/cytoplasmic staining with intensity of 2+/3+ and a distribution of 4+ (cases with at least moderate staining intensity in ≥75% of tumor cells) [31,32]. Cases with cytoplasmic staining alone were considered negative [33].

### 2.6. Statistical Analysis

Categorical variables were summarized using counts and percentages. The Chi-square test or Fisher’s exact test was used in a univariate study to discover relationships between p16^INK4a^ expression and clinicopathological features. The Kaplan–Meier method was used to estimate the OS and DFS, and the log-rank test was performed to compare the results. All statistical analyses were undertaken with a two-tailed test. The threshold for statistical significance was set at *p*-values less than or equal to 0.05. Statistical analysis was performed using IBM SPSS Statistics software for Windows version 29 (IBM Corp., New York, NY, USA).

## 3. Results

### 3.1. Patients’ Characteristics

Table 1 summarizes the primary features of the patients’ sample. The cohort included 100 NSCLC patients who underwent surgical resection. Most patients underwent a lobectomy (84/100, 84%), ten patients underwent wedge resection, and six patients had a pneumonectomy. Six patients had positive bronchial or vascular margins. In terms of their histologic subtype, 59% of the cases were ADC, and 41% were SqCC. Most patients were older than 60 with a median age of 69 (range 46–86 years), and 85% were males. The vast majority of patients (~85%) were either current or former smokers. Most patients had high-grade tumors with poor differentiation (59%). The majority of cases were of early stages, and the distribution of the pathological stages was as follows: stage I (33%), stage II (36%), stage III (28%) and stage IV (3%). Most patients had a tumor size of more than 3 cm (64%), and 37% had positive lymph node metastasis. Regarding the architectural patterns of the ADC cases, the majority had an acinar predominant pattern (47.5%), followed by solid (25.4%), lepidic (23.7%), and micropapillary (3.4%).

Regarding the postoperative treatment, 47 patients received no therapy, 38 patients received chemotherapy, 4 patients received radiotherapy, and 11 patients had a combination of chemoradiation therapy. About 34 patients developed disease recurrence or progression either in the form of locoregional recurrence or distant metastasis. Of these, four patients did not have any further therapy; nine patients had radiation therapy, five patients had chemotherapy, eight patients received chemoradiation therapy, three patients received a combination of chemotherapy with immunotherapy, one patient received a combination of chemotherapy and epidermal growth factor receptor (EGFR) tyrosine kinase inhibitor, one patient received EGFR tyrosine kinase inhibitor alone, one patient was treated with Anaplastic lymphoma kinase (ALK) inhibitor, one patient received immunotherapy, and one patient was treated with surgery alone. The status of the driver gene mutations was available for a limited number of cases. ALK1 was tested in 32 cases by an FDA-approved CDx grade IHC, and only 2 were positive. ROS1 was tested in six cases by IHC, and only one was positive. EGFR gene mutations were investigated in eleven cases, where only two cases showed exon 19 deletion and L858R mutation. The tumor proportion score of PD-L1 was available for 47 cases, where 28 cases were positive, with a score of 1% or more.

### 3.2. HPV Detection and Correlation with p16^INK4a^ Expression

**Figure 1:**
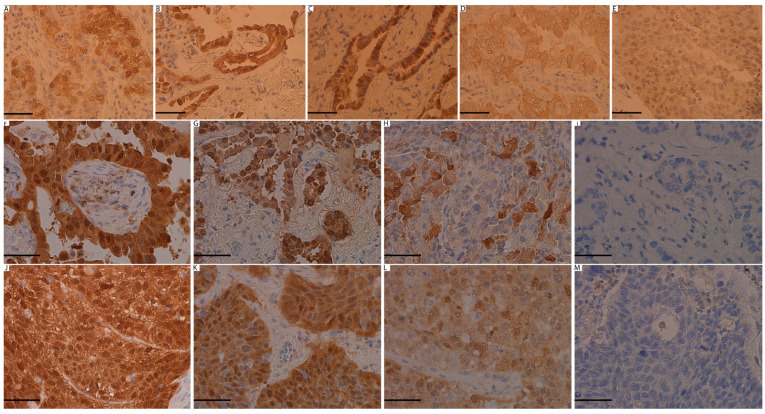
Representative images of p16^INK4a^ immunohistochemistry expression. (**A**) p16^INK4a^ expression in HPV-16-positive ADC case. (**B**) p16^INK4a^ expression in HPV-18-positive ADC case. (**C**) p16^INK4a^ expression in HPV-52-positive ADC case. (**D**) p16^INK4a^ expression in HPV-26-positive ADC case. (**E**) p16^INK4a^ expression in HPV-26-positive SqCC case. All HPV-positive cases showed negative p16^INK4a^ results with variable expression demonstrating weak to moderate intensity. (**F**) p16^INK4a^ expression showing score 3+ with strong nuclear and cytoplasmic expression in ADC. (**G**) p16^INK4a^ expression showing score 2+ with moderate nuclear and cytoplasmic expression in ADC. (**H**) p16^INK4a^ expression showing score 1+ with weak nuclear and cytoplasmic expression in ADC. (**I**) p16^INK4a^ expression showing score 0 with complete lack of staining in ADC. (**J**) p16^INK4a^ expression showing score 3+ with strong nuclear and cytoplasmic expression in SqCC. (**K**) p16^INK4a^ expression showing score 2+ with moderate nuclear and cytoplasmic expression in SqCC. (**L**) p16^INK4a^ expression showing score 1+ with weak nuclear and cytoplasmic expression in SqCC. (**M**) p16^INK4a^ expression showing a score of 0 with a complete lack of staining in SqCC. Abbreviations: ADC: adenocarcinoma, SqCC: squamous cell carcinoma. All images were obtained at 40× magnification. Scale bars = 5 µm.

Real-time PCR revealed that among the 100 NSCLC cases, 5 cases (5%) were positive for HPV. Three cases were positive for HR-HPV subtypes (16, 18, and 52), and two cases were positive for the probable HR-HPV 26 subtype (Table 2). Four of the positive cases were ADC, and only one case was of SqCC histological type. These positive cases were detected in three males and two females with a mean age of 70 (range: 56–78). Four cases occurred in patients with a smoking history and one in a non-smoker. Four of them also demonstrated low-grade disease, and one was high-grade. Three cases were of pathological stage I, 1 of stage II, and 1 of stage III. Only one of these positive cases showed a progression of the disease. The median follow-up time of the patients with HPV-positive tumors was 36 months (range: 24–70). Surprisingly, none of the HPV-positive cases were positive for p16^INK4a^ by IHC according to the followed criteria. However, all cases showed p16^INK4a^ expression ranging from 5 to 60% with weak to moderate intensity (Figure 1A–E). No statistically significant association was identified between the HPV status and other clinicopathological variables.

### 3.3. p16^INK4a^ Expression Detection and Correlation with the Clinicopathological Features

In total, 20 out of 100 cases were positive for p16^INK4a^ expression. Of those, 12 cases were ADC (12/59, 20.3%), and 8 cases were SqCC (8/41, 19.5%). In both ADC and SqCC, p16^INK4a^ expression varied from being strongly positive to aberrant to completely negative (Figure 1F–M). Cases with non-specific cytoplasmic staining were considered negative. Analysis of p16^INK4a^ expression in correlation to the clinicopathological variables revealed a statistically significant association with the pathological stage (*p =* 0.029), where most of the cases with positive p16^INK4a^ expression were either stage I or II. No significant association was demonstrated with the other characteristics, including HPV status (Table 1).

### 3.4. Survival Analysis of p16^INK4a^ Expression and Other Clinicopathological Variables

**Figure 2:**
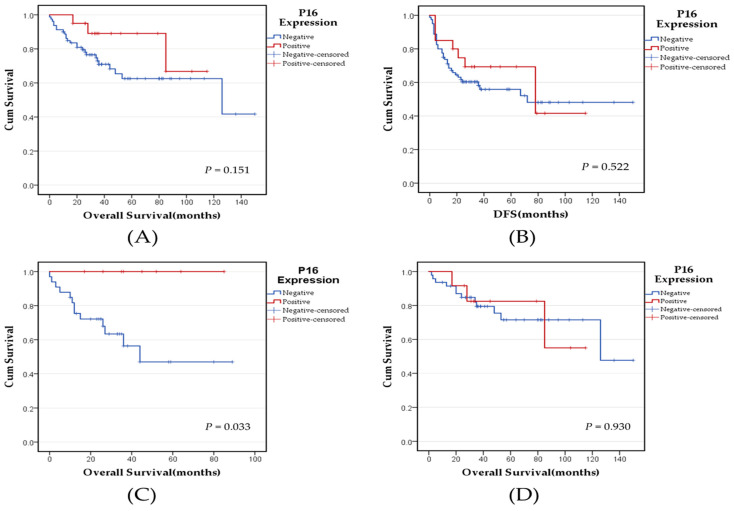
Kaplan–Meier survival curves according to p16^INK4a^ expression in NSCLC, ADC, and SqCC cases. (**A**) OS of p16^INK4a^-positive and negative NSCLC tumors. (**B**) DFS of p16^INK4a^-positive and negative NSCLC tumors. (**C**) OS of p16^INK4a^-positive and negative SqCC tumors. (**D**) OS of p16^INK4a^-positive and negative ADC tumors. Abbreviations: NSCLC: non-small-cell lung cancer, ADC: adenocarcinoma, SqCC: squamous cell carcinoma, OS: overall survival, DFS: disease-free survival. *p*-values of ≤0.05 were regarded as statistically significant.

**Figure 3:**
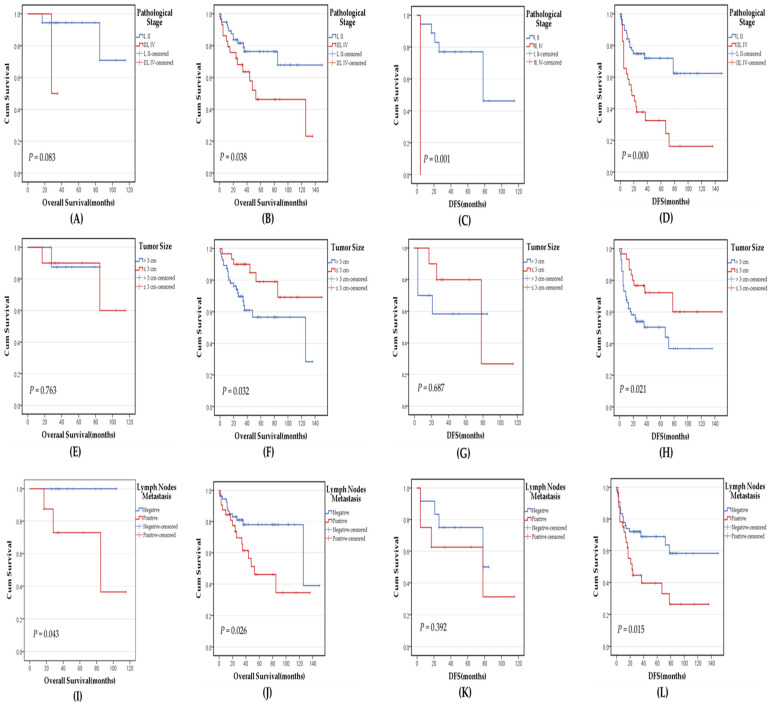
Kaplan–Meier survival curves of OS and DFS according to different clinicopathological variables based on p16^INK4a^ expression. (**A**,**B**) The correlation between the OS and different pathological tumor stages in p16^INK4a^-positive and negative cases, respectively. (**C**,**D**) The correlation between the DFS and different pathological tumor stages in p16^INK4a^-positive and negative cases, respectively. Tumor stages were divided into two groups, one composed of early stages I and II and another group comprised of late tumor stages III and IV. (**E**,**F**) The correlation between the OS and tumor sizes in p16^INK4a^-positive and negative cases, respectively. (**G**,**H**) The correlation between the DFS and tumor sizes in p16^INK4a^-positive and negative cases, respectively. (**I**,**J**) The correlation between the OS and lymph node metastasis status in p16^INK4a^-positive and negative cases, respectively. (**K**,**L**) The correlation between the DFS and lymph node metastasis status in p16^INK4a^-positive and negative cases, respectively. Abbreviations: OS: overall survival, DFS: disease-free survival. *p*-values of ≤0.05 were regarded as statistically significant.

The median overall survival (OS) for all patients was 35 months (mean 43.45 months), and the median disease-free survival (DFS) was 26.5 months (mean 36.11 months) following surgical resection. Survival analysis of all cases showed no statistically significant association between p16^INK4a^ expression and either OS (*p* = 0.151) or DFS (*p* = 0.522). However, by dividing the cases according to the histological subtypes, a statistically significant association was found between p16^INK4a^ expression and OS in SqCC cases, where cases with positive p16^INK4a^ expression exhibited a better overall survival (*p* = 0.033) but not in ADC cases (*p* = 0.930) (Figure 2A–D). Kaplan–Meier survival analysis showed that advanced pathological stages, positive lymph node metastasis, and tumor size > 3 cm were significantly associated with poor OS and DFS in p16^INK4a^ negative cases. In contrast, in p16^INK4a^ positive cases, only positive lymph node metastasis and advanced pathological stages were significantly associated with poor OS and DFS, respectively (Figure 3A–L).

## 4. Discussion

The role of HPV as a causative agent in lung cancer has been previously suggested but with wide variability based on geographic and methodological differences [34]. This work represents the first study to investigate the prevalence of HPV infection in NSCLC among a Jordanian subpopulation. This study investigated 100 NSCLC cases, particularly ADC and SqCC types, from a central referral hospital in Jordan. All cases were assessed for the presence of 12 HR-HPV, 6 probable HR-HPV, and 3 LR-HPV genotypes. HPV prevalence in this study was 5%; four cases were of the ADC histological subtype, and one was of the SqCC subtype. Three cases were of high-risk subtypes, and two cases were of the probable high-risk HPV 26 subtype (40%, 2/5). Due to the rarity of studies investigating the relationship between HPV infection and lung cancer, locally and in the neighboring countries in the Middle East and North Africa (MENA) area, it was challenging to compare our findings with previous work. A study conducted by Nadji et al. in Iran investigated the presence of HPV DNA in 129 lung cancer cases and 90 non-cancer control subjects using nested PCR. The study found an HPV prevalence of 25.6% in lung cancer cases compared to 9% in control cases, with HR-HPV 16 and 18 subtypes being more prevalent in cancer cases than in control cases [35]. Interestingly, similar to our results, they detected only one HPV-26-positive lung cancer case. Another more recent study in an Iranian subpopulation investigated a relatively similar sample size to the one in this work, including 109 lung cancer cases and 52 control cases [36]. The study utilized RT-PCR to analyze the presence of HPV, assessed the expression of E2, E6, and E7 viral oncoproteins, the expression of p53 and Rb genes, and selected miRNAs and genes related to epithelial–mesenchymal transition (EMT). They reported a 51.4% prevalence of HPV among lung cancer cases and 23.1% among control samples, which is significantly higher than what was observed by our analysis. HPV 16 was the most detected type, both in cancer and control cases. Of interest, authors also suggested that HPV infection could play a role in EMT [36].

The prevalence of HPV among lung cancer cases had a higher frequency in Asian populations compared to European and North American populations, as reported by several meta-analyses [4,37,38]. The HPV frequency range has been found to be 0 to 78.3% (mean 33.9%) in Asia, 0 to 69.2% (mean 10.5%) in Europe, 27.8% to 29% (mean 28.6%) in South America and 0 to 22% (mean 10.2%) in North America [39]. Although our results revealed a low prevalence of HPV positivity in NSCLC among the Jordanian population, it falls within the worldwide range of 0.0 to 78.3%, and it shows partial agreement with some studies [4]. For example, Joh et al. investigated 51 frozen lung samples from 30 NSCLC patients to assess the presence of HPV using PCR and DNA sequencing [40]. HPV DNA was identified in 16.7% of NSCLC patients, all of which were of ADC histological type, with HPV 16 being the most prevalent genotype [40]. Another study investigated 176 lung SqCC and 128 lung ADC from eight Asian institutions for the presence of HPV using PCR and in situ hybridization (ISH). HPV infection was detected in 6.3% and 7% of patients with lung SqCC and lung ADC, respectively. Most of the HPV-positive cases were HPV 16/18 genotypes [41]. Similarly, Sarchianaki et al. investigated 100 NSCLC using RT-PCT and reported a prevalence of 19% for HPV with a higher frequency in ADC cases [2], which is similar to the current study. On the other hand, Yanagawa et al. assessed the presence of the HPV genotype in 336 primary NSCLC cases using PCR and ISH and detected a prevalence of 1.5% using both methods. All cases were of the HPV 16 genotype and were found in SqCC [39]. Similarly, de Oliveira et al. reported a higher frequency of HPV in lung SqCC compared to ADC cases. Inconsistent with these results, Silva et al. investigated the presence of HPV DNA in 62 NSCLC cases using multiplex PCR and HPV16-specific RT-PCR, and none of the cases were positive for HPV [42]. Similar results of HPV negativity in lung cancer were reported in other two studies using different methods [43,44].

As suggested by previous studies, it seems that the heterogeneity in HPV prevalence is attributed to geographic differences, sociocultural differences, sensitivities of the methods used, sample size, and, finally, the histologic subtypes of the studied cases. Evidently, in Jordan, the prevalence of oncogenic HPV is relatively low in comparison to Western countries, with some studies reporting a general prevalence of 4% in a subpopulation of patients [45]. In this work, no statistically significant association was identified between the HPV status and other clinicopathological variables. This can be attributed to the very small number of HPV-positive cases detected. Similar to our results, some previous studies failed to find an association between HPV and other clinicopathological features in lung cancer [2,46,47].

p16^INK4a^ overexpression has a strong correlation with HR-HPV infection in uterine cervix carcinoma and a subset of oropharyngeal SqCC and is frequently utilized as a surrogate for such cancers [43]. However, this link has not been adequately established in lung cancer. Therefore, we further assessed the expression of p16^INK4a^ in the whole cohort of patients, trying to explore any correlation with the HPV status. p16^INK4a^ overexpression was detected in 20% of cases; surprisingly, none of these cases were positive for HPV DNA, and further, none of the five HPV-positive cases were positive for p16^INK4a^ overexpression considering at least a moderate intensity of staining in ≥75% of tumor cells as positive. Still, all HPV-positive cases showed some degree of positive p16^INK4a^ expression ranging from weak to moderate intensity. HPV-unrelated p16^INK4a^ overexpression can be attributed to Rb gene inactivation by mechanisms other than HR-HPV E7 expression [48,49] or be part of tumor cell senescence, as reported previously in lung cancer [50,51,52]. Similarly, several previous studies failed to find a correlation between p16^INK4a^ overexpression and HPV status in lung cancer [39,42,47]. For example, Bishop et al. assessed 220 cases of lung SqCC and found a prevalence of 24.5% of p16^INK4a^ overexpression, considering a strong and diffuse nuclear and cytoplasmic staining present in ≥70% of the tumor as positive, while only 5% of cases were positive for HR-HPV by ISH [21]. In agreement with that, Doxtader et al. reported a 35% positivity rate of p16^INK4a^ in lung SqCC (considering a strong and diffuse nuclear and cytoplasmic staining present in ≥50% of the tumor as positive), where all cases were negative for HR-HPV [15], and similarly considering a strong and diffuse nuclear and cytoplasmic staining present in ≥70% of the tumor as positive, Chang et al. reported a strong diffuse expression of p16^INK4a^ in 14.6% of NSCLC cases with negative results for HPV [43]. Subsequently, p16^INK4a^ expression might not be a reliable surrogate for HPV infection in lung cancer. On the contrary, Robinson et al. investigated 70 NSCLC cases where p16^INK4a^ expression significantly correlated with the presence of HPV [53]. Further, high p16^INK4a^ expression may have provided more persuasive evidence that the virus has molecularly affected cellular proliferation, according to some studies [53,54].

Regardless of the HPV status, the correlation between the p16^INK4a^ expression and other clinicopathological factors and its prognostic value in lung cancer is not clear. In this study, p16^INK4a^ expression was significantly correlated with early-stage disease. In agreement with this, Bian et al. reported that negative p16^INK4a^ expression by IHC has a statistically significant correlation with a higher pathological stage and lymph node metastasis but not with other variables such as age, gender, differentiation, and tumor size [55]. Another study revealed that p16^INK4a^ overexpression is significantly associated with early-stage IA1 and IA2 disease [25]. Another exciting study used the same scoring criteria of p16^INK4a^ expression as in this work and found that p16^INK4a^ positivity is significantly associated with the N0 stage, while p16^INK4a^ negativity is significantly associated with SqCC [24]. On the contrary, Zou et al. [47] and Silva et al. [42] did not find a significant correlation between p16^INK4a^ expression and other clinicopathological features.

When all cases of NSCLC were analyzed, no significant impact of the p16^INK4a^ expression on the OS (*p* = 0.151) or DFS (*p* = 0.522) was detected in this work. However, p16^INK4a^ expression showed a statistically significant association with OS in patients with the SqCC histological subtype, where patients with positive p16^INK4a^ expression showed a better overall survival (*p* = 0.033). Further, poor OS and DFS were significantly associated with advanced pathological stages, positive lymph node metastasis, and tumor size >3 cm in p16^INK4a^ negative cases, while, in p16^INK4a^-positive cases, only positive lymph node metastasis and advanced pathological stages were significantly associated with poor OS and DFS, respectively. Similarly, Huang et al. did not find a significant difference in five-year survival and p16^INK4a^ status in NSCLC; however, a worse OS rate of patients with negative p16^INK4a^ was established in early stages [33]. In contrast, Zhou et al. found that p16^INK4a^ expression was associated with a statistically significant favorable prognosis in ADC cases, whereas in SqCC, p16^INK4a^ expression showed a slightly worse median OS, albeit not statistically significant [47]. Bian et al. also reported that loss of p16^INK4a^ expression, tumor size, lymph node metastasis, and pathological stages are associated with significantly shorter OS in lung ADC [55]. On the other hand, An et al. indicated that negative p16^INK4a^ expression was significantly associated with shorter disease-specific survival and DFS, and similar to our results, advanced pathological tumor stages were significantly associated with poor survival [24]. Collectively, the evidence supports a prognostic role of p16^INK4a^ expression in lung cancer and the variability between different studies can be attributed to different methodological approaches, especially p16^INK4a^ scoring, different sample sizes, and different histological subtypes.

This study has some limitations. First, this retrospective study was performed in a single center and, thus, was amenable to selection bias. Second, it utilized a single modality to assess the presence of HPV in spite of the fact that PCR is a sensitive and specific method to investigate HPV in lung cancer. Further, we recognize that the mere presence of HPV in lung cancer does not provide sufficient evidence about its role in carcinogenesis, and this requires further investigations. Finally, the relatively small sample size may have affected the results.

## 5. Conclusions

This is the first study to assess the HPV status and p16^INK4a^ expression in lung cancer among a Jordanian subpopulation and one of the very few conducted in the MENA area. This study showed that HPV is rare in NSCLC among the Jordanian population, with a prevalence in the studied sample of 5%, all of the high-risk or probable high-risk groups. Although p16^INK4a^ expression was detected in 20% of cases, unlike other cancer types, its reliability as a surrogate for HPV infection in lung cancer requires further investigation. p16^INK4a^ expression carries a good prognostic value in SqCC lung cancer but not in ADC cases. Overall, the potential role of HPV and p16^INK4a^ in the carcinogenesis of lung cancer should be further studied.

## Figures and Tables

**Table 1:** Patients’ clinicopathological features according to p16^INK4a^ expression.

Variables	Number (%)	p16^INK4a^ Positive ^b^	p16^INK4a^ Negative	*p* Value *
Total	100 (100.0)	20 (20.0)	80 (80.0)	
Age (Years)				0.232
≤60	23 (23.0)	7 (35.0)	16 (20.0)	
>60	77 (77.0)	13 (65.0)	64 (80.0)	
Gender				0.072
Male	85 (85.0)	14 (70.0)	71 (88.7)	
Female	15 (15.0)	6 (30.0)	9 (11.3)	
Smoking history ^a^				0.729
Current/former smoker	84 (84.8)	16 (80.0)	68 (86.1)	
Never smoker	15 (15.2)	4 (20.0)	11 (13.9)	
Histological subtype				1.000
Adenocarcinoma (ADC)	59 (59.0)	12 (60.0)	47 (58.8)	
Squamous cell carcinoma (SqCC)	41 (41.0)	8 (40.0)	33 (41.2)	
Grade				0.617
Low-grade (well and moderately differentiated)	41 (41.0)	7 (35.0)	34 (42.5)	
High-grade (poorly differentiated)	59 (59.0)	13 (65.0)	46 (57.5)	
Tumor size				0.193
≤3 cm	36 (36.0)	10 (50.0)	26 (32.5)	
>3 cm	64 (64.0)	10 (50.0)	54 (67.5)	
Lymph nodes metastasis				0.799
Positive	37 (37.0)	8 (40.0)	29 (36.3)	
Negative	63 (63.0)	12 (60.0)	51 (63.7)	
Pathological stage				**0.029**
I, II	69 (69.0)	18 (90.0)	51 (63.7)	
III, IV	31 (31.0)	2 (10.0)	29 (36.3)	
Predominant histological pattern (For ADC cases, 59 cases)				0.422
Lepidic	14 (23.7)	3 (25.0)	11 (23.4)	
Acinar	28 (47.5)	4 (33.3)	24 (51.1)	
Micropapillary	2 (3.4)	1 (8.3)	1 (2.1)	
Solid	15 (25.4)	4 (33.3)	11 (23.4)	
Papillary	0 (0.0)	0 (0.0)	0 (0.0)	
Recurrence/Progression				1.000
Present	34 (34.0)	7 (35.0)	27 (33.8)	
Absent	66 (66.0)	13 (65.0)	53 (66.3)	
HPV Status				
Positive	5 (5.0)	0 (0.0)	5 (6.3)	0.580
Negative	95 (95.0)	20 (100.0)	75 (93.7)	

^a^ One patient was excluded due to the absence of smoking history. ^b^ p16^INK4a^ was considered positive by immunohistochemistry when it showed at least moderately strong nuclear and cytoplasmic staining in ≥75% of tumor cells. * 2-sided *p*-value, *p*-value ≤ 0.05 is considered significant (bold).

**Table 2:** Clinicopathological characteristics of HPV-positive tumors.

HPV-Positive Tumor	Age	Gender	Smoking History	Histological Subtype	Grade ^a^	Pathological Stage	Recurrence/Progression	p16^INK4a^ Expression ^b^
HPV 16+	56	Female	Former smoker	ADC	High	Stage I	Absent	Negative
HPV 18+	74	Male	Smoker	ADC	Low	Stage II	Present	Negative
HPV 26+	72	Male	Smoker	SqCC	Low	Stage I	Absent	Negative
HPV 26+	78	Male	Smoker	ADC	Low	Stage III	Absent	Negative
HPV 52+	70	Female	Non-smoker	ADC	Low	Stage I	Absent	Negative

^a^ Low-grade tumors include well- and moderately differentiated tumors. High-grade tumors include poorly differentiated tumors. ^b^ P16 was considered positive by immunohistochemistry when it showed at least moderately strong nuclear and cytoplasmic staining in ≥75% of tumor cells. Abbreviations: ADC: adenocarcinoma, SqCC: squamous cell carcinoma.

## Data Availability

The data collected and analyzed during the current study are available upon reasonable request from the corresponding author.

## Abbreviations

HPV	Human papillomavirus
HR-HPV	High-risk human papillomavirus
NSCLC	Non-small-cell lung cancer
IHC	Immunohistochemistry
FFPE	Formalin-fixed, paraffin-embedded
KHCC	King Hussein Cancer Center
RT-PCR	Real-time polymerase chain reaction
AJCC	American Joint Committee on Cancer protocol
ADC	Adenocarcinoma
SqCC	Squamous cell carcinoma
LR-HPV	Low-risk human papillomavirus
CDK	Cyclin-dependent kinase
WHO	World Health Organization
OS	Overall survival
DFS	Disease-free survival
EGFR	Epidermal growth factor receptor
ALK	Anaplastic lymphoma kinase
ISH	In situ hybridization

## References

[1] Torre L.A., Bray F., Siegel R.L., Ferlay J., Lortet-Tieulent J., Jemal A. (2015). Global Cancer Statistics, 2012. CA Cancer J. Clin..

[2] Sarchianaki E., Derdas S.P., Ntaoukakis M., Vakonaki E., Lagoudaki E.D., Lasithiotaki I., Sarchianaki A., Koutsopoulos A., Symvoulakis E.K., Spandidos D.A. (2014). Detection and Genotype Analysis of Human Papillomavirus in Non-Small Cell Lung Cancer Patients. Tumor Biol..

[3] Sun S., Schiller J.H., Gazdar A.F. (2007). Lung Cancer in Never Smokers—A Different Disease. Nat. Rev. Cancer.

[4] Srinivasan M., Taioli E., Ragin C.C. (2009). Human Papillomavirus Type 16 and 18 in Primary Lung Cancers—A Meta-Analysis. Carcinogenesis.

[5] Spyratos D., Zarogoulidis P., Porpodis K., Tsakiridis K., Machairiotis N., Katsikogiannis N., Kougioumtzi I., Dryllis G., Kallianos A., Rapti A. (2013). Occupational Exposure and Lung Cancer. J. Thorac. Dis..

[6] de Freitas A.C., Gurgel A.P., de Lima E.G., de França São Marcos B., do Amaral C.M.M. (2016). Human Papillomavirus and Lung Cancinogenesis: An Overview. J. Cancer Res. Clin. Oncol..

[7] Zhai K., Ding J., Shi H.-Z. (2015). HPV and Lung Cancer Risk: A Meta-Analysis. J. Clin. Virol..

[8] Bruni L., Albero G., Serrano B., Mena M., Collado J.J., Gómez D., Muñoz J., Bosch F.X., de Sanjosé S. ICO/IARC Information Centre on HPV and Cancer (HPV Information Centre). Human Papillomavirus and Related Diseases in the World. Summary Report 10 March 2023. https://hpvcentre.net/statistics/reports/XWX.pdf.

[9] Corneanu L.M., Stănculescu D., Corneanu C. (2011). HPV and Cervical Squamous Intraepithelial Lesions: Clinicopathological Study. Rom. J. Morphol. Embryol..

[10] Berman T.A., Schiller J.T. (2017). Human Papillomavirus in Cervical Cancer and Oropharyngeal Cancer: One Cause, Two Diseases. Cancer.

[11] Muñoz N., Castellsagué X., de González A.B., Gissmann L. (2006). Chapter 1: HPV in the Etiology of Human Cancer. Vaccine.

[12] Gillison M.L., Koch W.M., Capone R.B., Spafford M., Westra W.H., Wu L., Zahurak M.L. (2000). Evidence for a Causal Association Between Human Papillomavirus and a Subset of Head and Neck Cancers. J. Natl. Cancer Inst..

[13] Preti M., Boldorini R., Gallio N., Cavagnetto C., Borella F., Pisapia E., Ribaldone R., Bovio E., Bertero L., Airoldi C. (2024). Human papillomavirus genotyping in high-grade vaginal intraepithelial neoplasia: A multicentric Italian study. J. Med. Virol..

[14] Sano T., Oyama T., Kashiwabara K., Fukuda T., Nakajima T. (1998). Immunohistochemical Overexpression of P16 Protein Associated with Intact Retinoblastoma Protein Expression in Cervical Cancer and Cervical Intraepithelial Neoplasia. Pathol. Int..

[15] Doxtader E.E., Katzenstein A.-L.A. (2012). The Relationship between P16 Expression and High-Risk Human Papillomavirus Infection in Squamous Cell Carcinomas from Sites Other than Uterine Cervix: A Study of 137 Cases. Hum. Pathol..

[16] Khleif S.N., DeGregori J., Yee C.L., Otterson G.A., Kaye F.J., Nevins J.R., Howley P.M. (1996). Inhibition of Cyclin D-CDK4/CDK6 Activity Is Associated with an E2F-Mediated Induction of Cyclin Kinase Inhibitor Activity. Proc. Natl. Acad. Sci. USA.

[17] Munger K., Jones D.L. (2015). Human Papillomavirus Carcinogenesis: An Identity Crisis in the Retinoblastoma Tumor Suppressor Pathway. J. Virol..

[18] McLaughlin-Drubin M.E., Crum C.P., Münger K. (2011). Human Papillomavirus E7 Oncoprotein Induces KDM6A and KDM6B Histone Demethylase Expression and Causes Epigenetic Reprogramming. Proc. Natl. Acad. Sci. USA.

[19] Cheng Y.-W., Wu M.-F., Wang J., Yeh K.-T., Goan Y.-G., Chiou H.-L., Chen C.-Y., Lee H. (2007). Human Papillomavirus 16/18 E6 Oncoprotein Is Expressed in Lung Cancer and Related with P53 Inactivation. Cancer Res..

[20] Syrjänen K., Syrjänen S., Kellokoski J., Kärjä J., Mäntyjärvi R. (1989). Human Papillomavirus (HPV) Type 6 and 16 DNA Sequences in Bronchial Squamous Cell Carcinomas Demonstrated by in Situ DNA Hybridization. Lung.

[21] Bishop J.A., Ogawa T., Chang X., Illei P.B., Gabrielson E., Pai S.I., Westra W.H. (2012). HPV Analysis in Distinguishing Second Primary Tumors From Lung Metastases in Patients With Head and Neck Squamous Cell Carcinoma. Am. J. Surg. Pathol..

[22] Myong N.-H. (2008). Cyclin D1 Overexpression, P16 Loss, and pRb Inactivation Play a Key Role in Pulmonary Carcinogenesis and Have a Prognostic Implication for the Long-Term Survival in Non-Small Cell Lung Carcinoma Patients. Cancer Res. Treat..

[23] Sterlacci W., Tzankov A., Veits L., Zelger B., Bihl M.P., Foerster A., Augustin F., Fiegl M., Savic S. (2011). A Comprehensive Analysis of P16 Expression, Gene Status, and Promoter Hypermethylation In Surgically Resected Non-Small Cell Lung Carcinomas. J. Thorac. Oncol..

[24] An H.J., Koh H.M., Song D.H. (2019). New P16 Expression Criteria Predict Lymph Node Metastasis in Patients With Non-Small Cell Lung Cancer. Vivo.

[25] Pezzuto A., Cappuzzo F., D’Arcangelo M., Ciccozzi M., Navarini L., Guerrini S., Ricci A., D’Ascanio M., Carico E. (2020). Prognostic Value of P16 Protein in Patients With Surgically Treated Non-Small Cell Lung Cancer; Relationship With Ki-67 and PD-L1. Anticancer Res..

[26] Qaqish A., Abdo N., Abbas M.M., Saadeh N., Alkhateeb M., Msameh R., Tarawneh S., Al-Masri M. (2023). Awareness and Knowledge of Physicians and Residents on the Non-Sexual Routes of Human Papilloma Virus (HPV) Infection and Their Perspectives on Anti-HPV Vaccination in Jordan. PLoS ONE.

[27] Travis W.D., Brambilla E., Nicholson A.G., Yatabe Y., Austin J.H.M., Beasley M.B., Chirieac L.R., Dacic S., Duhig E., Flieder D.B. (2015). The 2015 World Health Organization Classification of Lung Tumors. J. Thorac. Oncol..

[28] Nicholson A.G., Tsao M.S., Beasley M.B., Borczuk A.C., Brambilla E., Cooper W.A., Dacic S., Jain D., Kerr K.M., Lantuejoul S. (2022). The 2021 WHO Classification of Lung Tumors: Impact of Advances Since 2015. J. Thorac. Oncol..

[29] Mirsadraee S., Oswal D., Alizadeh Y., Caulo A., van Beek E.J. (2012). The 7th Lung Cancer TNM Classification and Staging System: Review of the Changes and Implications. World J. Radiol..

[30] Detterbeck F.C. (2018). The Eighth Edition TNM Stage Classification for Lung Cancer: What Does It Mean on Main Street?. J. Thorac. Cardiovasc. Surg..

[31] Würdemann N., Wagner S., Sharma S.J., Prigge E.-S., Reuschenbach M., Gattenlöhner S., Klussmann J.P., Wittekindt C. (2017). Prognostic Impact of AJCC/UICC 8th Edition New Staging Rules in Oropharyngeal Squamous Cell Carcinoma. Front. Oncol..

[32] Machczyński P., Majchrzak E., Niewinski P., Marchlewska J., Golusiński W. (2020). A Review of the 8th Edition of the AJCC Staging System for Oropharyngeal Cancer According to HPV Status. Eur. Arch. Otorhinolaryngol..

[33] Huang C.-I., Taki T., Higashiyama M., Kohno N., Miyake M. (2000). P16 Protein Expression Is Associated with a Poor Prognosis in Squamous Cell Carcinoma of the Lung. Br. J. Cancer.

[34] Syrjänen K. (2012). Detection of Human Papillomavirus in Lung Cancer: Systematic Review and Meta-Analysis. Anticancer Res..

[35] Nadji S.A., Mokhtari-Azad T., Mahmoodi M., Yahyapour Y., Naghshvar F., Torabizadeh J., Ziaee A.A., Nategh R. (2007). Relationship between Lung Cancer and Human Papillomavirus in North of Iran, Mazandaran Province. Cancer Lett..

[36] Hussen B.M., Ahmadi G., Marzban H., Fard Azar M.E., Sorayyayi S., Karampour R., Nahand J.S., Hidayat H.J., Moghoofei M. (2021). The Role of HPV Gene Expression and Selected Cellular MiRNAs in Lung Cancer Development. Microb. Pathog..

[37] Klein F., Amin Kotb W.F.M., Petersen I. (2009). Incidence of Human Papilloma Virus in Lung Cancer. Lung Cancer.

[38] Hasegawa Y., Ando M., Kubo A., Isa S., Yamamoto S., Tsujino K., Kurata T., Ou S.-H.I., Takada M., Kawaguchi T. (2014). Human Papilloma Virus in Non-Small Cell Lung Cancer in Never Smokers: A Systematic Review of the Literature. Lung Cancer.

[39] Yanagawa N., Wang A., Kohler D., Santos G.D.C., Sykes J., Xu J., Pintilie M., Tsao M.-S. (2013). Human Papilloma Virus Genome Is Rare in North American Non-Small Cell Lung Carcinoma Patients. Lung Cancer.

[40] Joh J., Jenson A.B., Moore G.D., Rezazedeh A., Slone S.P., Ghim S., Kloecker G.H. (2010). Human Papillomavirus (HPV) and Merkel Cell Polyomavirus (MCPyV) in Non Small Cell Lung Cancer. Exp. Mol. Pathol..

[41] Goto A., Li C.-P., Ota S., Niki T., Ohtsuki Y., Kitajima S., Yonezawa S., Koriyama C., Akiba S., Uchima H. (2011). Human Papillomavirus Infection in Lung and Esophageal Cancers: Analysis of 485 Asian Cases. J. Med. Virol..

[42] Silva E.M., Mariano V.S., Pastrez P.R.A., Pinto M.C., Nunes E.M., Sichero L., Villa L.L., Scapulatempo-Neto C., Syrjanen K.J., Longatto-Filho A. (2019). Human Papillomavirus Is Not Associated to Non-Small Cell Lung Cancer: Data from a Prospective Cross-Sectional Study. Infect. Agent. Cancer.

[43] Chang S.Y., Keeney M., Law M., Donovan J., Aubry M.-C., Garcia J. (2015). Detection of Human Papillomavirus in Non–Small Cell Carcinoma of the Lung. Hum. Pathol..

[44] Iwakawa R., Kohno T., Enari M., Kiyono T., Yokota J. (2010). Prevalence of Human Papillomavirus 16/18/33 Infection and P53 Mutation in Lung Adenocarcinoma. Cancer Sci..

[45] Khasawneh A.I., Asali F.F., Kilani R.M., Abu-Raideh J.A., Himsawi N.M., Salameh M.A., Al Ghabbiesh G.H., Saleh T. (2020). Prevalence and Genotype Distribution of Human Papillomavirus Among a Subpopulation of Jordanian Women. Int. J. Womens Health.

[46] Ragin C., Obikoya-Malomo M., Kim S., Chen Z., Flores-Obando R., Gibbs D., Koriyama C., Aguayo F., Koshiol J., Caporaso N.E. (2014). HPV-Associated Lung Cancers: An International Pooled Analysis. Carcinogenesis.

[47] Zhou Y., Höti N., Ao M., Zhang Z., Zhu H., Li L., Askin F., Gabrielson E., Zhang H., Li Q.K. (2019). Expression of P16 and P53 in Non-Small-Cell Lung Cancer: Clinicopathological Correlation and Potential Prognostic Impact. Biomark. Med..

[48] Gorgoulis V.G., Zacharatos P., Kotsinas A., Liloglou T., Kyroudi A., Veslemes M., Rassidakis A., Halazonetis T.D., Field J.K., Kittas C. (1998). Alterations of the P16-pRb Pathway and the Chromosome Locus 9p21–22 in Non-Small-Cell Lung Carcinomas. Am. J. Pathol..

[49] Romagosa C., Simonetti S., López-Vicente L., Mazo A., Lleonart M.E., Castellvi J., Ramon Y Cajal S. (2011). p16Ink4a Overexpression in Cancer: A Tumor Suppressor Gene Associated with Senescence and High-Grade Tumors. Oncogene.

[50] Paez-Ribes M., González-Gualda E., Doherty G.J., Muñoz-Espín D. (2019). Targeting Senescent Cells in Translational Medicine. EMBO Mol. Med..

[51] Domen A., Deben C., De Pauw I., Hermans C., Lambrechts H., Verswyvel J., Siozopoulou V., Pauwels P., Demaria M., Van De Wiel M. (2022). Prognostic Implications of Cellular Senescence in Resected Non-Small Cell Lung Cancer. Transl. Lung Cancer Res..

[52] Saleh T., Bloukh S., Hasan M., Al Shboul S. (2023). Therapy-Induced Senescence as a Component of Tumor Biology: Evidence from Clinical Cancer. Biochim. Biophys. Acta Rev. Cancer.

[53] Robinson L.A., Jaing C.J., Pierce Campbell C., Magliocco A., Xiong Y., Magliocco G., Thissen J.B., Antonia S. (2016). Molecular Evidence of Viral DNA in Non-Small Cell Lung Cancer and Non-Neoplastic Lung. Br. J. Cancer.

[54] Marcos B.F.S., De Oliveira T.H.A., Do Amaral C.M.M., Muniz M.T.C., Freitas A.C. (2022). Correlation between HPV PCNA, P16, and P21 Expression in Lung Cancer Patients. Cell. Microbiol..

[55] Bian C., Li Z., Xu Y., Wang J., Xu L., Shen H. (2015). Clinical Outcome and Expression of Mutant P53, P16, and Smad4 in Lung Adenocarcinoma: A Prospective Study. World J. Surg. Oncol..

